# Impaired autophagic flux and its related inflammation in patients with adult-onset Still’s disease

**DOI:** 10.18632/oncotarget.23098

**Published:** 2017-12-11

**Authors:** Chia-Wei Hsieh, Chun-Yu Chang, Yi-Ming Chen, Hsin-Hua Chen, Wei-Ting Hung, Ning-Rong Gung, Shiow-Jiuan Wey, Der-Yuan Chen

**Affiliations:** ^1^ Ph.D. Program in Translational Medicine and Rong Hsing Research Center for Translational Medicine, National Chung Hsing University, Taichung, Taiwan; ^2^ Division of Allergy, Immunology and Rheumatology, Taichung Veterans General Hospital, Taichung, Taiwan; ^3^ Department of Medical Education and Research, Taichung Veterans General Hospital, Taichung, Taiwan; ^4^ Faculty of Medicine, National Yang Ming University, Taipei, Taiwan; ^5^ Division of Dermatology, Chung-Shan Medical University Hospital, Taichung, Taiwan

**Keywords:** autophagy, autophagy-related genes, autophagic flux, inflammation, adult-onset Still’s disease, Immunology

## Abstract

The pathogenic role of autophagic immune regulation in adult-onset Still’s disease (AOSD) is unclear. We investigated the relative levels of autophagy in AOSD patients and healthy controls, its association with disease activity or course, and the change in autophagy after 6 months of therapy. Autophagosome levels were determined from the mean fluorescence intensity of autophagosomotropic dye incorporated into circulating immune cells. The fluorescent signal from lymphocytes, monocytes, and granulocytes from AOSD patients was greater than from controls. Levels of p62 fluorescence measured using flow cytometry in lymphocytes and granulocytes from AOSD patients was greater than in the corresponding cells from healthy controls. Expression of Atg5 and LC3-II mRNA and protein levels of p62 and LC3-II were elevated in AOSD patients. Moreover, AOSD activity scores correlated positively with autophagosome levels in monocytes and granulocytes, p62 levels in circulating immune cells, and levels of Beclin-1, Atg5, and LC3-II mRNA. Autophagosome levels and Atg mRNA expression decreased with disease remission in AOSD patients. Elevated autophagosome formation and p62 levels suggest impaired autophagic flux in AOSD.

## INTRODUCTION

Autophagy is the process of engulfment and degradation of cytoplasmic contents by lysosomes [1-2]. After autophagy induction, cytoplasmic vesicle nucleation occurs through formation of the mammalian orthologue of autophagy-related gene 6 (Atg6)/type III phosphatidylinositol 3-kinase (PIK3III)/Atg14 complex, Beclin-1. The autophagophore membrane elongates and closes to form an autophagosome through the conjugation of light chain three (LC3) to phosphatidylethanolamine through the Atg5/Atg12/Atg16L complex [1-3]. LC3 consists of a soluble form (LC3I, molecular weight 18 kDa) and a lipidated form (LC3-II, 16 kDa). The LC3-binding adaptor protein p62 (SQSTM1/sequestosome-1) binds ubiquitinated substrates, serves as a molecular bridge for delivery to autophagosomes, and then promotes degradation through a proteasomal pathway [4-5]. Finally, the autophagosome fuses with a lysosome to form an autolysosome that digests the engulfed cargo [1-3]. The expression of Atg and p62 is commonly used to monitor autophagic activity and flux.

Tight regulation of autophagy is crucial for preventing uncontrolled activation and for inflammatory responses [1, 6-9], including the clearance of protein complexes such as inflammasomes through proteasomal degradation [10]. The networks between autophagy and inflammation are complex. Autophagy is involved in the induction and suppression of inflammation, and vice versa [6-9]. Proinflammatory cytokines such as interleukin (IL)-1β and IL-18 enhance autophagosome formation in macrophages [11-12]. Autophagy influences IL-1β secretion by regulating inflammasome activation [10]. Due to the multifaceted roles of autophagy in inflammatory responses [6-10], dysregulated autophagy has been implicated in the pathogenesis of autoimmune diseases and autoinflammatory diseases [13-16].

Adult-onset Still’s disease (AOSD) is a systemic inflammatory disease characterized by fever, rash, arthritis, multisystemic involvement, and increased acute phase reactants [17-18]. It is now regarded as an autoinflammatory disease due to phenotypical signatures and the absence of a significant increase in autoantibody levels [19]. While the pathogenesis of AOSD remains elusive, the disease is characterized by increased levels of proinflammatory cytokines such as IL-1β and IL-18 [20-22]. We recently demonstrated the elevated NLRP3 (NOD-like receptor containing pyrin domain 3)-inflammasome expression in AOSD patients and found that imiquimod was a potential activator of NLRP3-inflammasome that upregulated expression [23]. A recent report revealed that imiquimod possessed anti-cancer effects against melanoma via autophagic cell death [24]. Given the associations of proinflammatory cytokines and NLRP3-inflammasome with AOSD, and between inflammation and autophagy, we hypothesized that autophagy might have an important role in AOSD pathogenesis. We compared the autophagosome and p62 levels in circulating immune cells, as well as the Atg mRNA and protein expression of AOSD patients and healthy controls (HC). We investigated the association of autophagy expression with disease activity parameters, clinical manifestations, or disease course in AOSD patients and the changes in autophagy expression after 6-month therapy.

## RESULTS

### Clinical characteristics of AOSD patients

Among the 28 AOSD-active patients, fever ≥39 °C (100%), rash (92.9%), arthritis (50.0%), lymphadenopathy (46.4%), liver dysfunction (42.9%), and hepatosplenomegaly (25.0%) detected by abdominal sonography was noted. There were no significant differences in the age at entry (mean age ± SD, 35.6 ± 8.8 years *vs*. 37.0 ± 12.5 years) or the proportion of females (23/28, 82.1% *vs*. 17/20, 85.0%) between AOSD patients and HCs.

### Mean fluorescence intensity of Cyto-ID in circulating lymphocytes, monocytes, and granulocytes

**Figure 1:**
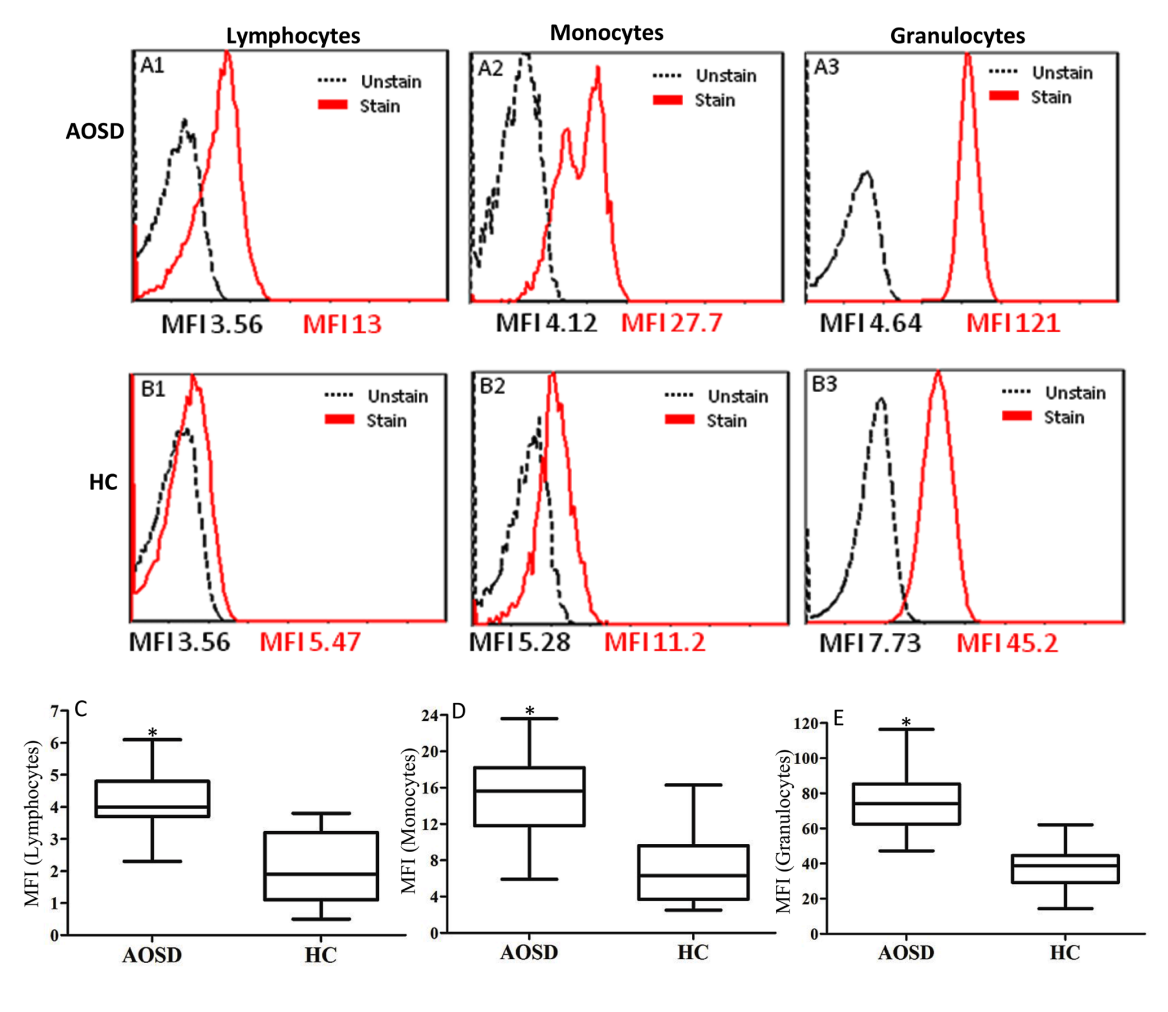
Representative cytometric histograms from one AOSD patient (A) and one HC (B) of Cyto-ID-staining in lymphocytes (A1 and B1), monocytes (A2 and B2), and granulocytes (A3 and B3) Comparisons of autophagosome levels of Cyto-ID-staining in lymphocytes **C.**, monocytes **D.**, and granulocytes **E.** between AOSD patients and HCs. Data are presented as box-plot diagrams, with the box encompassing the 25^th^ percentile (lower bar) to the 75^th^ percentile (upper bar). The horizontal line within the box indicates median value respectively for each group. *p < 0.001, *vs*. HC.

Autophagy was detected by the autophagosome-specific CytoID tracer dye and quantified by flow cytometry [25]. The representative cytometric histograms of Cyto-ID-staining obtained from one AOSD patient and one HC subject are shown in Figure 1A-1B. Significantly higher mean fluorescence intensity (MFI) was observed in circulating lymphocytes, monocytes, and granulocytes from AOSD patients (median 4.14, interquartile range [IQR] 3.68-5.03; 15.75, 13.58-20.22; 78.32, 68.07-86.62; respectively) compared with HCs (median 2.02, IQR 1.12-3.71; 7.49, 3.74-12.41; 41.08, 32.52-61.99; respectively, all *p* < 0.001, Figure 1C-1E).

### MFI of p62 in circulating lymphocytes, monocytes, and granulocytes

**Figure 2:**
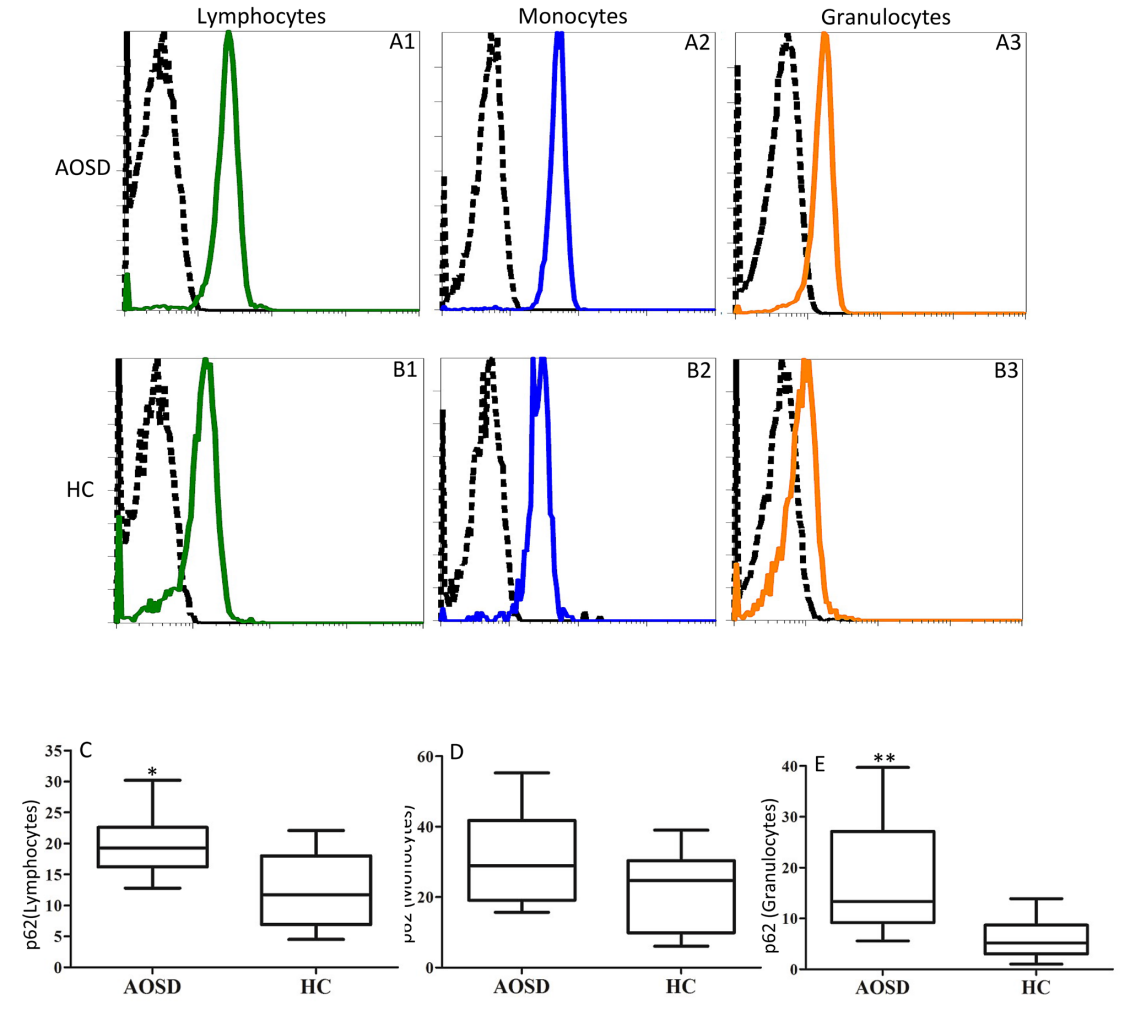
Representative cytometric histograms of p62 levels in lymphocytes (A1 and B1), monocytes (A2 and B2), and granulocytes (A3 and B3) from one AOSD patient **A.** one HC **B.** Comparisons of the MFI of p62 in lymphocytes **C.**, monocytes **D.** and granulocytes **E.**, between AOSD patients and HCs. Data are presented as box-plot diagrams, with the box encompassing the 25^th^ percentile (lower bar) to the 75^th^ percentile (upper bar). The horizontal line within the box indicates median value for each group. *p < 0.005, **p < 0.001, *vs*. HC.

The representative examples of cytometric histograms of p62 levels obtained from one AOSD patient and one HC subject are shown in Figure 2A-2B. Significantly higher values of MFI of p62 were observed in circulating lymphocytes and granulocytes from AOSD patients (median 19.25, IQR 16.18-22.60; 13.30, 9.2-27.13; respectively) compared with HCs (11.70, 6.90-18.03, *p* < 0.005; 5.20, 3.03-8.65, *p* < 0.001; respectively, Figure 2C and 2E). However, there was no difference in the MFI of p62 in circulating monocytes.

### The mRNA expression levels of autophagy-related genes and serum cytokines levels

**Figure 3:**
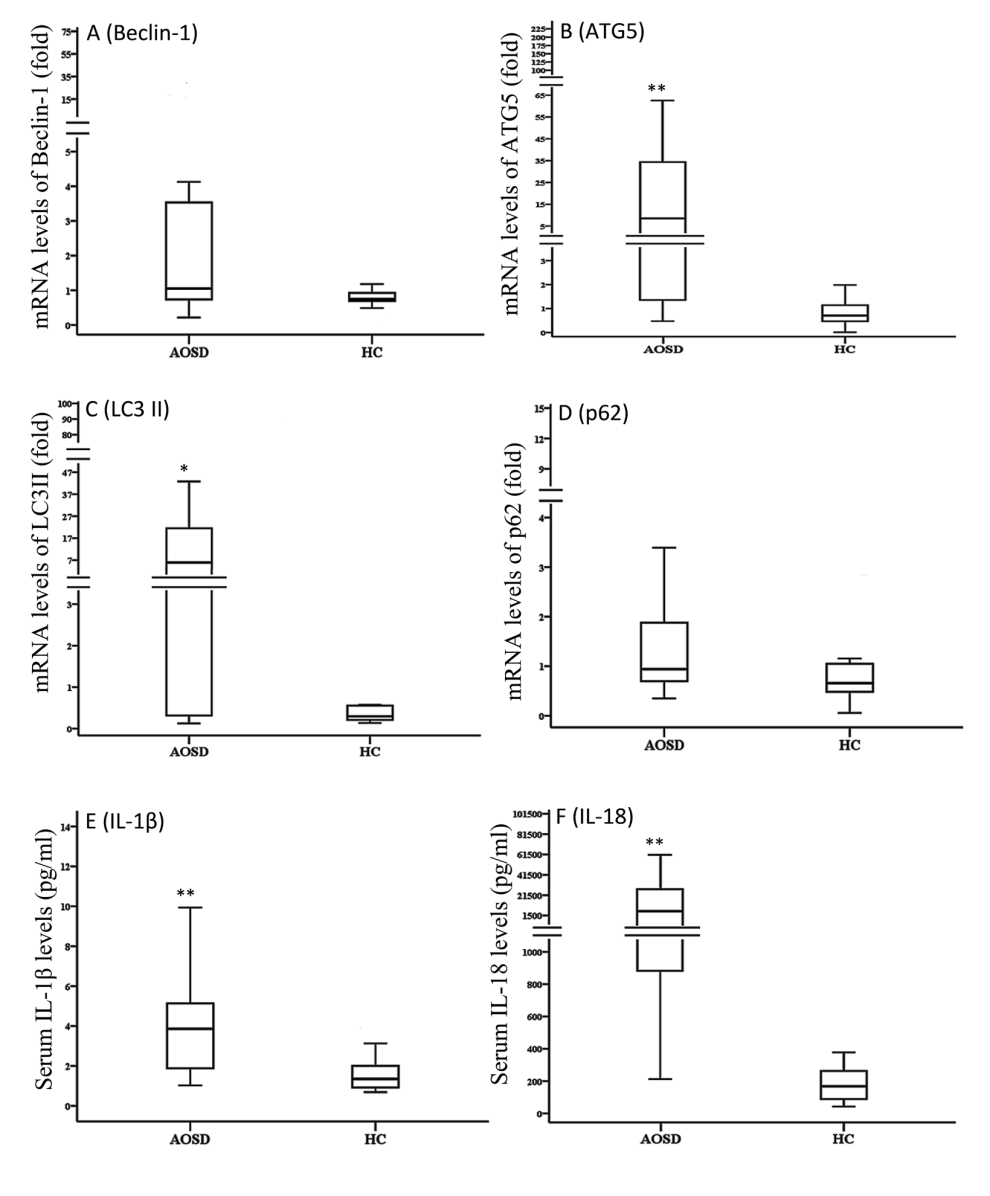
Comparisons of the mRNA expression levels of autophagy-related genes, including Beclin-1 **A.**, Atg5 **B.**, LC3-II **C.**, and p62 **D.** Serum levels of IL-1β **E.** and IL-18 **F.** between AOSD patients and HCs. LC3-II: microtubule-associated protein one light chain three-II. Data are presented as box-plot diagrams, with the box encompassing the 25^th^ percentile (lower bar) to the 75^th^ percentile (upper bar). The horizontal line within the box indicates median value for each group. *p < 0.01, **p < 0.001, *vs*. HC.

As shown in Figure 3, significantly higher mRNA expression levels of Atg5 and LC3-II (MAP1LC3B) were observed in AOSD patients (median 8.53, IQR 1.35-36.60; 5.86, 0.30-23.08; respectively) than in HCs (0.71, 0.481.21, *p* < 0.001; 0.31, 0.22-0.58, *p* < 0.01; respectively). Significantly higher median levels of serum cytokines, IL-1β and IL-18, were observed in AOSD-active patients (median 3.86 pg/mL, IQR 1.83-5.20 pg/mL; 5757.5 pg/mL, 824.3-27657.0 pg/mL; respectively) compared with HCs (1.37 pg/mL, 0.87-2.15 pg/mL; 167.1 pg/mL, 79.5-297.2 pg/mL; respectively, both *p* < 0.001).

### Atg protein expression levels in AOSD patients and HC

**Figure 4:**
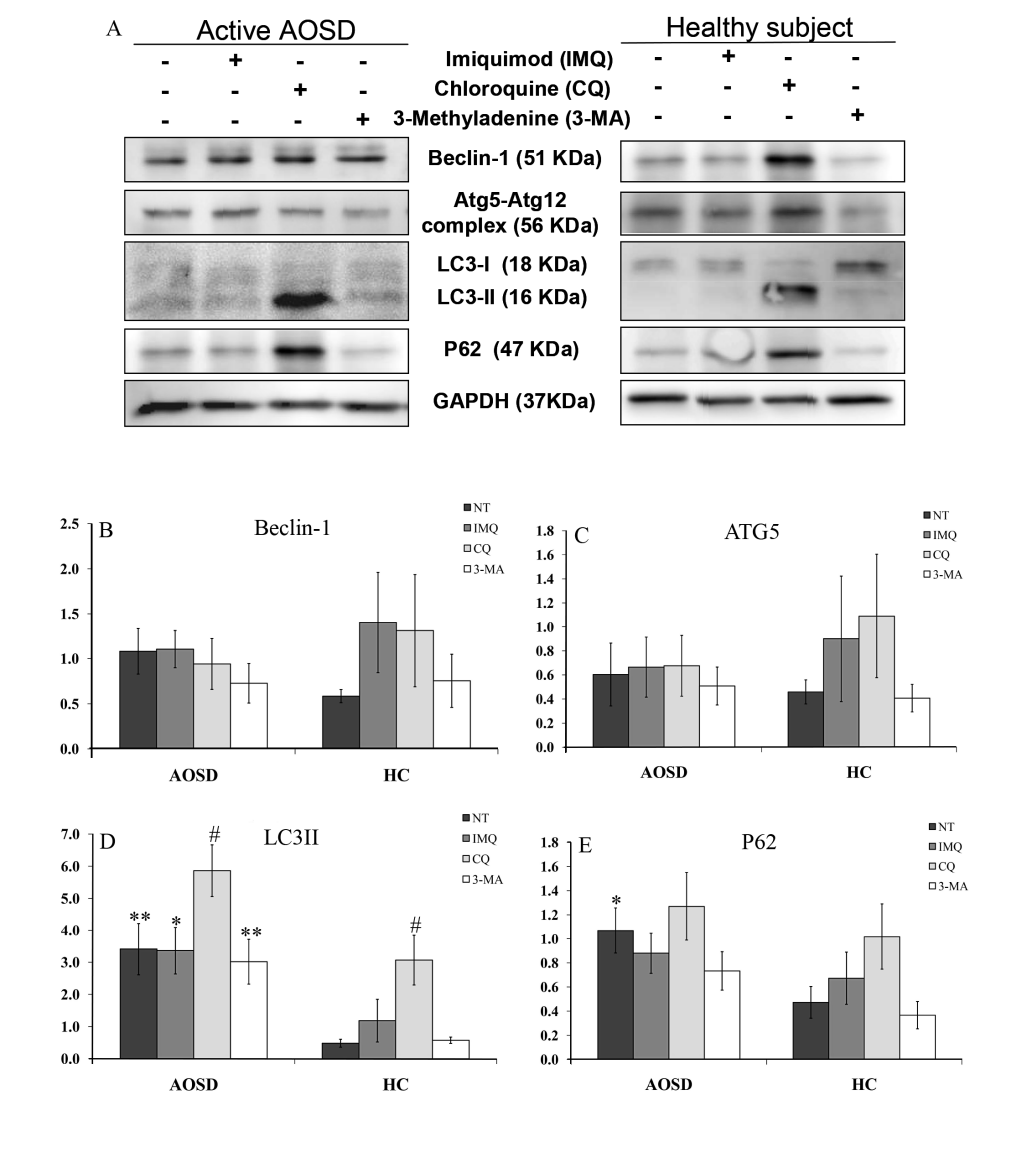
The effects of potential activator or inhibitor on protein expression levels of autophagy-related molecules in PBMCs **A.** Representative examples of Atg protein expression in PBMC lysates treated with imiquimod, chloroquine, and 3-methyladenine in one AOSD patient and one HC. The fold change in protein levels of **B.** Beclin-1, **C.** Atg5, **D.** LC3-II, and p62 **E.** in AOSD patients and HCs. Bars and error bars indicate the mean and standard error of the mean, respectively. *p < 0.05, **p < 0.005, *vs*. HC; #*p* < 0.05, *vs*. no treatment group or 3-methyladenine-treated group, as determined by Mann-Whitney U test.

Representative immunoblotting analyses of Atg expression in PBMC lysates were obtained from one AOSD patient and one HC (Figure 4A). Protein expression of LC3-II and p62 in AOSD-active patients (mean ± SEM, 3.4 ± 0.8 and 1.1 ± 0.2, respectively) were significantly higher than HCs (0.5 ± 0.1, *p* < 0.005 and 0.5 ± 0.1, *p* < 0.05, respectively). However, there were no significant differences in Beclin-1 or Atg5 protein expression in PBMC lysates between AOSD patients and HC.

Autophagic flux was analyzed by chloroquine treatment to determine whether increased LC3-II levels resulted from reduced autophagic degradation. As shown in Figure 4, chloroquine treatment further enhanced LC3-II protein expression in PBMCs of AOSD patients (3.4 ± 0.8 *vs*. 5.9 ± 0.8, *p* < 0.05) and HCs (0.5 ± 0.1 *vs*. 3.1 ± 0.8, *p* < 0.05). Although statistical significance was not reached, chloroquine treatment enhanced p62 protein expression of in PBMCs from AOSD patients (1.1 ± 0.8 *vs*. 1.3 ± 0.3) and HCs (0.5 ± 0.1 *vs*. 1.0 ± 0.3) (Figure 4E). There was no significant change in Atg protein expression in PBMCs after treatment with imiquimod or 3-methyladenine in AOSD patients or HCs.

### Correlation between autophagy expression and inflammatory parameters in AOSD

As illustrated in Table 1, AOSD activity scores and ferritin levels were positively correlated with the autophagosome levels in monocytes or granulocytes as evidenced by the MFIs of Cyto-ID and p62 in circulating immune cells. Disease activity scores were also significantly correlated with the mRNA levels of Beclin-1, Atg5, and LC3-II. Serum IL-1β levels were positively correlated with autophagosome levels in circulating monocytes or granulocytes and LC3-II mRNA expression. Serum IL-18 levels were positively correlated with autophagosome levels in circulating granulocytes.

**Table 1:** Correlations between autophagy expression levels and disease activity parameters or serum levels of proinflammatory cytokines in 28 patients with adult-onset Still’s disease (AOSD)

Autophagy expression levels	Activity scores	Ferritin levels	IL-1β levels	IL-18 levels
MFI in lymphocytes	0.061	0.163	0.250	0.054
MFI in monocytes	0.422*	0.422*	0.632**	0.233
MFI in granulocytes	0.501*	0.412*	0.461*	0.532*
P62 in lymphocytes	0.594**	0.438*	0.303	0.275
P62 in monocytes	0.408*	0.508**	0.359	0.218
P62 in granulocytes	0.502*	0.176	0.174	0.157
mRNA levels of Beclin-1	0.561**	0.400	0.054	0.253
mRNA levels of Atg5	0.600**	0.280	0.280	0.309
mRNA levels of LC3-II	0.544**	0.321	0.475*	0.353
mRNA levels of p62	0.139	0.032	0.002	0.219

MFI: mean fluorescence intensity; Atg5: autophagy-related gene protein 5; LC3-II: microtubule-associated protein one light chain three-II; IL-1β: interleukin-1β; IL-18: interleukin-18.

*p<0.05, **p<0.01, were determined by Spearman’s rank correlation test

### Logistic regression analysis

Logistic regression analysis was performed to evaluate the simultaneous autophagy expression levels and clinical manifestations in AOSD patients. The autophagosome levels in circulating monocytes were identified as a significant predictor of liver dysfunction or hepatosplenomegaly (Odds ratio [OR] 1.31, 95% confidence interval [95% CI] 1.02-1.69, *p* = 0.037; 1.27, 1.01-1.60, *p* = 0.041; respectively).

### Differential autophagy expression and disease course in AOSD patients

Among AOSD patients, 9 (32.1%) had a monocyclic pattern, and 19 (67.9%) had a polycyclic pattern of disease course. The p62 MFI values in circulating lymphocytes and monocytes of AOSD patients with a polycyclic pattern (median 22.0, IQR 17.2-23.0; 32.0, 26.8-42.5; respectively) were higher than those with a monocyclic pattern (17.8, 14.8-18.8; 18.8, 17.8-30.0; respectively, both *p* < 0.05). Significantly lower p62 MFI values in granulocytes were found in patients with a polycyclic pattern compared to those with a monocyclic pattern (11.1, 7.7-15.0 *vs*. 30.1, 12.4-36.2, *p* < 0.05). There were no differences in the autophagosome levels or Atg mRNA expression between patients with polycyclic and monocyclic patterns.

### Modified autophagy expression in AOSD patients after 6-month therapy

**Figure 5:**
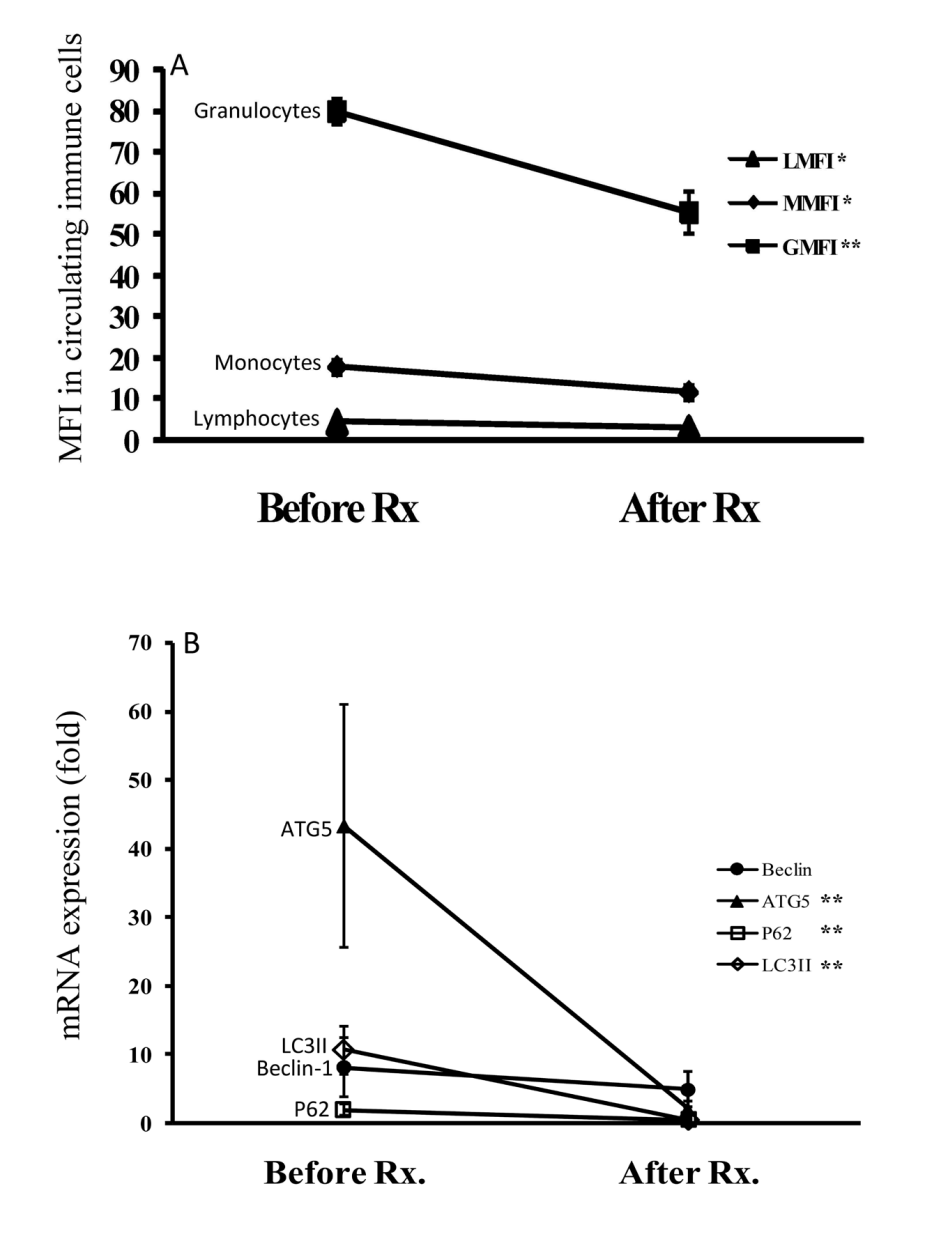
The changes in (A) autophagosome levels evidenced by Cyto-ID MFI in circulating immune cells and (B) the mRNA levels of autophagy-related genes including Beclin-1, Atg5, LC3-II, and p62 after 6-month therapy in AOSD patients Data are presented as the mean ± standard error of the mean. *p < 0.05, **p < 0.01, *vs*. before treatment, as determined by Wilcoxon signed rank test.

As illustrated in Figure 5, the autophagosome levels of circulating lymphocytes, monocytes, and granulocytes significantly decreased (mean ± SEM, 4.4 ± 0.3 *vs*. 3.1 ± 0.4, *p* < 0.05; 17.7 ± 1.8 *vs*. 11.6 ± 1.7, *p* < 0.05; 79.8 ± 3.1 *vs*. 55.3 ± 5.1, *p* < 0.005; respectively) in AOSD patients after 6-month treatment. Similarly, mRNA expression levels of Atg5, p62, and LC3-II declined significantly (43.31 ± 17.76 *vs*. 2.08 ± 1.15, *p* < 0.05; 1.98 ± 0.78 *vs*. 0.52 ± 0.09, *p* < 0.05; 10.68 ± 3.50 *vs*. 0.52 ± 0.26, *p* < 0.05; respectively) and paralleled the decrease in disease activity score (4.83 ± 0.32 *vs*. 2.56 ± 0.25, *p* < 0.01). There was no significant change in Beclin-1 mRNA expression in AOSD patients.

## DISCUSSION

Dysregulated autophagy is associated with inflammatory diseases [8, 14-16], but the involvement of autophagy in AOSD pathogenesis is unclear. We demonstrated increased autophagosome levels in circulating immune cells from AOSD patients compared to HCs. The mRNA and protein expression levels of LC3-II, which is indicative of autophagosome formation, were also elevated in AOSD patients. Higher p62 levels indicated impairment of autophagic degradation in AOSD patients as compared to HC. The combination of elevated autophagosome formation and p62 levels suggested the involvement of impaired autophagic flux in AOSD pathogenesis. However, larger prospective studies are needed to confirm these results.

Autophagosome formation is a critical step in the process of autophagy [1-2]. Previous studies have revealed elevated autophagy in other inflammatory diseases [14-16]. Significantly higher levels of autophagosome formation in circulating immune cells as well as increased LC3-II mRNA and protein expression were observed in AOSD-active patients compared with HCs. These results are consistent with reports of the activated autophagic process in systemic lupus erythematosus [26-28], which shares partial clinical features with AOSD. Furthermore, autophagosome levels significantly decreased after treatment, paralleling disease remission in AOSD patients. These results supported a recent report of reduced basal autophagy following disease remission in patients with familial Mediterranean fever [29].

We examined the Atg mRNA and protein expression levels in PBMCs of AOSD patients and HCs to evaluate the regulation of autophagosome formation. Atg5, which regulating autophagic elongation, and LC3-II, indicative of autophagosome formation [1-2], expression was higher in AOSD-active patients compared to HCs. LC3-II protein levels increased further in PBMCs treated with the autophagic flux inhibitor, chloroquine [30]. Our findings suggest that autophagosome formation is normal or upregulated in AOSD. We did not observe a significant change in Atg protein expression in PBMCs after treatment with the potential autophagy activator, imiquimod [24]. This discrepancy might be related to differences in disease characteristics or imiquimod dosage.

The positive correlation between autophagosome levels and inflammatory parameters, including disease activity score and serum levels of IL-1β or IL-18, suggested that elevated autophagosome formation levels were associated with inflammation in AOSD patients. IL-1β triggers autophagy in macrophages and IL-18 has been demonstrated to stimulate autophagy [11-12]. Cytokine-induced inflammation may be regulated by a negative feedback mechanism [10, 31]. The causative effect of proinflammatory cytokines on autophagy activation in AOSD should be evaluated by future studies.

The p62 protein is selectively degraded by autophagy [32]. The p62-bound ubiquitinated substrates are incorporated into the autophagosome and degraded into autolysosomes, and p62 serves as a readout of autophagic flux [4-5]. Decreased p62 levels are associated with autophagic process activation, while elevation reflects impairment of autophagic flux or autophagosomal degradation [33-34]. The p62 MFI values in circulating immune cells and protein expression in PBMCs of AOSD patients were increased compared to HCs. Chloroquine treatment upregulated LC3-II and p62 protein levels, which were attributed to accumulated autophagosomes and decreased autophagic flux, respectively. Impaired autophagic flux in AOSD patients may lead to the insufficient removal of damaged or activated macromolecules, such as inflammasomes or cytokines [10, 35], and contribute to inflammation. This hypothesis was supported by increased p62 levels in circulating lymphocytes and monocytes from AOSD patients that presented a polycyclic pattern compared to those with a monocyclic disease course pattern. Another recent study demonstrated that impaired autophagy flux and the resultant inadequate clearance of tumor necrosis factor receptor 1 (TNFR1) were implicated in patients with TNFR-associated periodic syndrome [16], an autoinflammatory disease.

Clinical manifestations are heterogeneous in AOSD patients [17-18]. Our logistical analysis revealed that autophagosome levels in circulating monocytes were a significant predictor of liver dysfunction or hepatosplenomegaly. These results supported a recent study that revealed accumulated autophagosomes and decreased autophagic flux in septic mice with liver injury [36].

Our pilot study had several limitations. The absence of a significant effect of 3-methyladenine might be related to the small number of the enrolled AOSD patients or the dual role of 3-methyladenine in the modulation of autophagy [37]. Medications, such as corticosteroids, may influence autophagy by reducing cytokine secretion [38] and drug interference should be considered. Further studies are needed to elucidate the mechanism of impaired autophagy flux in AOSD.

Elevated autophagosome formation and Atg expression were positively correlated with disease activity parameters, which suggested an association between autophagy and inflammation in AOSD. Increased p62 levels reflected decreased degradation during autophagic flux [4-5, 33-34]. The combination of increased autophagosome formation and p62 levels in AOSD patients indicated impaired autophagic flux. Autophagy promotion is a potential therapeutic modality, which has been used to protect against liver injury [39]. Our novel insights are of translational interest and could provide promising therapeutic targets.

## MATERIALS AND METHODS

### Subjects

In this prospective, monocentric study, 28 active AOSD patients fulfilling the Yamaguchi criteria were consecutively enrolled [40]. Patients with infections, malignancies, or other rheumatic diseases were excluded. Disease activity of AOSD was assessed using a modified Pouchot score described by Rau et al. [41]. Active AOSD was defined by a disease activity score of at least 3. All patients received corticosteroid treatment. After the initial investigation, 25 (89.3%) patients received at least one synthetic disease-modifying anti-rheumatic drugs (sDMARDs) including methotrexate (23), hydroxychloroquine (18), azathioprine (8), or cyclosporine (5). The definition of disease course was modified from previous studies [40, 42]. A monocyclic pattern was characterized by a unique/ self-limited course. A polycyclic pattern was characterized by recurrent flares of systemic and articular symptoms during a follow-up period of at least one year. Healthy volunteers (20) with no rheumatic disease were enrolled as control subjects. Venous blood samples were obtained in the morning, centrifuged at 1000 xg for 10 min within 15 min of withdrawal. All serum samples were stored at -70 °C until determination of proinflammatory cytokine levels. This study (CE14349A-1) was approved by the Institutional Review Board of the hospital, and written consent was obtained from each participant.

### Quantification of autophagosome levels in circulating immune cells by Cyto-ID

Fluorescence of autophagosomotropic dye, Cyto-ID, in circulating immune cells was measured using Cyto-ID™ Autophagy Detection Kit (Enzo Life Sciences, PA, USA) according to the manufacturer’s protocol and the described technique [26, 43-44]. Briefly, 100 μL of whole blood was stained with 0.25 μL/mL of Cyto-ID Green Autophagy Detection Reagent (Enzo Life Sciences, PA, USA) and 20 μL of Phycoerythrin-Cyanin 5 (PC5)-conjugated CD45-specific monoclonal antibody (mAb) (Beckman Coulter, Indianapolis IN, USA). After incubation for 30 min in the dark at room temperature, cells were reacted with OptiLyse Solution (Beckman Coulter, Indianapolis IN, USA) for 10 min to lyse red blood cells. After PBS washing, cells were analyzed by flow cytometry (Beckman Coulter, Brea, CA, USA). Monocytes, lymphocytes, and granulocytes were gated by CD45+/side scatters. At least 5 x 10^4^ total cells from each sample were analyzed. The gated lymphocytes, monocytes, and granulocytes were verified as follows: 100 μL samples of whole blood stained with 20 μL of fluorescein isothiocyanate (FITC)-conjugated CD3-specific mAb (Beckman Coulter, USA), 20 μL of PC5-conjugated CD14-specific mAb, and 20 μL of FITC-conjugated CD66b-specific mAb, respectively, with 20 μL of PC5-conjugated CD45-specific mAb separately for 15 min at room temperature. Data were expressed as the MFI of Cyto-ID.

### Quantification of p62 levels by flow cytometry

Intracellular immunofluorescence staining of p62 was performed following fixation and permeabilization using a modified previous method [26]. Briefly, 50 μL of whole blood was stained with 20 μL of FITC-conjugated CD45-specific mAb for 15 min at room temperature. Cells were fixed by adding 100 μL of Reagent 1 (Beckman Coulter, USA) for 15 min before centrifugation for 5 min at 300 xg. After supernatant removal, 100 μL of reagent 2 (Beckman Coulter, Brea, CA, USA) was added for permeabilization (10 min). Cells were subsequently incubated with peridinin chlorophyll protein (PerCP)-conjugated p62/SQSTM1 mAb (clone 5H7E2, Novus biological, Littleton, USA) for 15 min in the dark at room temperature. PerCP-conjugated IgG1 (R&D Systems, Minneapolis, N, USA) was used as the isotype control. Cells were immediately analyzed using flow cytometry (Beckman Coulter, USA).

### Determination of mRNA expression of autophagy-related genes by qPCR

Total RNA was obtained from peripheral blood mononuclear cells (PBMCs) by the guanidinium isothiocyanate method [45]. A 2.5 μg RNA aliquot was reverse transcribed using 200 U of Moloney murine leukemia virus reverse transcriptase (Fermentas, Thermo Fisher Scientific Inc., Pittsburgh, PA, USA). The Atg mRNA expression levels, including Beclin-1, Atg5, p62, and LC3-II, were determined by Roche FastStart Universal SYBR Green Master Mix (Roche Life Science, Indianapolis IN, USA). The primer sequences were as follows: Beclin-1, 5’-CCA TGCAGGTGAGCTTCGT-3’ (forward) and 5’-GAATCTGCGAGAGACACCATC-3’ (reverse); ATG5, 5’-AAAGATGTGCTTCGAGATGTGT-3’ (forward) and 5’-CACTTTGTCAGTTACCAACGTCA-3’(reverse); p62, 5’-AAGCCGGGTGG GAATGTTG-3’ (forward) and 5’-GCTTGGCCCTTCGGATTCT-3’ (reverse); LC3II (MAP1LC3B), 5’-AAGGCGCTTACAGCTCAATG-3’ (forward) and 5’-CTG GGAGGCATAGACCATGT-3’ (reverse); and GAPDH, 5’-GAAGGTGAAGGT CGGAGTC-3’ (forward) and 5’-GAAGATGGTGATGGGATTTC-3’ (reverse). The mRNA levels of the housekeeping gene GAPDH were determined for each sample to standardize the mRNA expression levels of autophagy-related molecules. Atg mRNA expression was calculated using comparative threshold cycle (Ct) method and evaluated by 2-^△△Ct^, where △△Ct =Patient (Ct_Atg_-Ct_GAPDH_) - Mean of HC (Ct_Atg_-Ct_GAPDH_).

### Determination of Atg protein expression using western blotting analysis

Total proteins were extracted from PBMC lysates treated with or without a potential autophagy activator (imiquimod) [24], an autophagic process inhibitor (3-methyladenine; Sigma-Aldrich, St. Louis, MO, USA) [46], and an autophagic flux inhibitor (chloroquine; Sigma-Aldrich, St. Louis, MO, USA) [30] for 6 hours at 37 °C in 5% CO_2_. The proteins were separated with 10-12% SDS-PAGE and then transferred to PVDF membranes (Bio-Rad, Hercules, CA, USA).

Immunoblots were performed using primary antibodies (1:1000 dilutions) overnight at 4 °C against Beclin-1 (Abcam, Cambridge, MA, USA), Atg5/Atg12/Atg16L complex (Abcam, Cambridge, MA, USA), p62 (Abcam, Cambridge, MA , USA ), LC3II (Abcam, Cambridge, MA, USA), and GAPDH (Santa Cruz Biotechnology, Dallas, Texas, USA), followed by incubation with HRP-conjugated anti-rabbit secondary antibody (1:5000) for 1 h at 37 °C (Santa Cruz Biotechnology, Dallas, Texas, USA). Image processing and data quantification were performed using Multi Gauge v2.02 software (Fujifilm). Atg protein expression levels were normalized to GAPDH.

### Determination of serum levels of proinflammatory cytokines using ELISA

Serum IL-1β levels were determined using ELISA (Ray Biotech Inc., Norcross, GA, USA) according to the manufacturer’s instruction. IL-18 levels were determined by ELISA (Medical and Biological Lab. CO., Nagoya, Aichi, Japan).

### Statistical analysis

Data were presented as the mean ± standard deviation or standard error of the mean (SEM), or median with the interquartile range (IQR). The Mann-Whitney U test was used for intergroup comparison of autophagy expression and cytokine levels. The correlation coefficient was obtained by Spearman’s rank test. A logistic regression analysis was performed to evaluate the effects of autophagy expression on clinical manifestation occurrence. The Wilcoxon signed rank test was used to evaluate changes in autophagy expression. A *p*-value < 0.05 was considered significant.
